# Supplementary material for: Heterogeneity in responses to ribosome-targeting antibiotics mediated by bacterial RNA repair
Source: Nat Commun. 2025 Nov 11;16:9620. doi: 10.1038/s41467-025-64759-3 (PMC12606134; doi:10.1038/s41467-025-64759-3)
Supplement: Supplementary file 1 — Supplementary Information [file 41467_2025_64759_MOESM1_ESM.pdf]

Supplementary information
Heterogeneity in responses to ribosome-targeting antibiotics mediated by bacterial RNA repair

Hollie J. Hindley[1], Zechuan Gong[2], Shafagh Moradian[2], Maria Grazia Giuliano[2,3], Andrei Sapelkin[4], Ioly Kotta-Loizou[5,6], Martin Buck[5], Christoph Engl[2,*], and Andrea Y. Weiße[1,7,*]

[1]Centre for Engineering Biology, School of Biological Sciences, University of Edinburgh, UK
[2]Department of Biochemistry, Centre for Molecular Cell Biology, School of Biological and Behavioural Sciences, Queen Mary University of London, UK
[3]Current address: Health Science Interdisciplinary Center, Sant'anna School of Advanced Studies, Pisa, Italy
[4]Department of Physics and Astronomy, School of Physical and Chemical Sciences, Queen Mary University of London, UK
[5]Department of Life Sciences, Faculty of Natural Sciences, Imperial College London, UK
[6]Department of Clinical, Pharmaceutical and Biological Science, School of Health, Medicine and Life Sciences, University of Hertfordshire, UK
[7]School of Informatics, University of Edinburgh, UK
[*]To whom correspondence should be addressed: c.engl@qmul.ac.uk, andrea.weisse@ed.ac.uk

Contents

S1 Model definitions **S3**
S1.1 The *rtc* operon . . . S3
S1.2 Rtc-regulated RNA repair . . . S4
S1.3 Comparison of predictions between rRNA and tRNA repair . . . S5
S1.4 Model extensions . . . S5
S1.4.1 Inhibition of Rtc proteins . . . S5
S1.4.2 Dynamic dilution and influx rates . . . S5
S1.4.3 Direct repair via RtcB only . . . S6

S2 Supporting Methods **S6**
S2.1 Quantification of *rtc* expression with qPCR . . . S6
S2.2 Parameterisation . . . S7
S2.3 Sensitivity analysis . . . S8
S2.4 RNA FISH probe design . . . S8
S2.5 Materials . . . S9

List of Figures

S1 Direct repair via RtcB only . . . S11
S2 Steady-state responses of the tRNA-repair model . . . S12
S3 Additional analysis of ribosome repair . . . S13
S4 Additional analysis of tRNA repair . . . S14
S5 The cyclisation of 2'-phosphate RNA termini by RtcA gives a bistable response in extreme parameter regimes. . . . S15
S6 Sensitivity analysis of bistability in the rRNA-repair model . . . S16
S7 Sensitivity analysis of bistability in the tRNA-repair model . . . S17
S8 Steady-state responses for ribosome repair with dynamic dilution and influx . . . S18
S9 Negative control for the smFISH experiment . . . S19
S10 Inhibiting Rtc proteins is predicted to reduce levels of resistance (tRNA repair) . . . S20

S11 Individual gene knockouts of the *rtc* genes do not impact ribosome levels in untreated cells. S21
S12 Measured and fitted Raman spectra. S21

List of Tables

S1 Model parameters. S7
S2 Parameters for Rtc inhibition S7
S3 Parameters for dynamic dilution & ribosome influx rates S8
S4 qPCR data used for tuning maximal transcription rates S8
S5 Probe sequences of RNA probes designed complementary to the *rtcB* gene sequence. . . . S9
S6 Materials detailing manufacturer and catalogue number. S10

S1 Model definitions

The Rtc model considers the expression and healing and sealing action of RtcA and RtcB respectively on three distinct species of translational RNA, which may represent rRNA within a ribosome or tRNA. We consider expression of the three *rtc* genes, the regulator *rtcR* and the co-expressed *rtcBA* gene. The model includes nine species in total, three mRNA species, m_x, three protein species, p_x and three translational RNA species, r_h, r_d, r_t for healthy, damaged and tagged (with cyclic RNA ends) RNA:

$$\dot{m}_x = v_{\text{tx}}^{(x)} - m_x \cdot (\lambda + d_m), \tag{S1}$$

$$\dot{p}_x = v_{\text{tl}}^{(x)} - \lambda \cdot p_x \qquad x \in A, B, R, \tag{S2}$$

$$\dot{r}_h = k_{\text{in}} + v_{\text{rep}} - r_h \cdot (\lambda + k_{\text{dam}}), \tag{S3}$$

$$\dot{r}_d = r_h \cdot k_{\text{dam}} - v_{\text{tag}} - r_d \cdot (\lambda + k_{\text{deg}}), \tag{S4}$$

$$\dot{r}_t = v_{\text{tag}} - v_{\text{rep}} - \lambda \cdot r_t. \tag{S5}$$

Here, x denotes the *rtc* gene, i.e. $x \in \{a, b, r\}$ for *rtcA, rtcB* and *rtcR*, $v_{\text{tx}}^{(x)}$ represents the overall transcription rates to mRNAs (Eq. (S8), (S15)) and $v_{\text{tl}}^{(x)}$ represents the translation rates to proteins (Eq. (7), (8)). Healthy RNA influx occurs at a constant rate k_{in}, v_{rep} represents the rate of repair by RtcB (Eq. (S19)) and v_{tag} represents the rate of tagging by RtcA (Eq. (S18)). All species are diluted due to growth by rate λ, mRNAs and damaged RNAs are degraded at rates of d_m and k_{deg} respectively and healthy RNAs are damaged at a rate of k_{dam} (Table S1).

S1.1 The *rtc* operon

The overall transcription and translation rates depend upon the elongation rates of mRNA and protein formation, respectively. Elongation requires energy and therefore we consider the rates to be ATP-dependent processes with Michaelis-Menten kinetics:

$$v_{\text{tx:elong}} = \frac{\omega_x \cdot \text{ATP}}{\theta_{\text{tx}} + \text{ATP}}, \qquad x \in \text{ba, r}, \tag{S6}$$

$$v_{\text{tl:elong}} = \frac{\gamma_{\max} \cdot \text{ATP}}{\theta_{\text{tl}} + \text{ATP}}, \tag{S7}$$

where ω_x and $\gamma_{\max}$ are the maximal transcription and translation rates, θ_{tx} and θ_{tl} are the corresponding transcription and translation thresholds, and ATP is the concentration of ATP in μM. Refer to Table S1 for full definitions and values.

We assume that *rtcR* is constitutively expressed. Its overall transcription rate is therefore equivalent to the rate of elongation (Eq. (S6))

$$v_{\text{tx}}^{(\text{R})} = \frac{\omega_{\text{r}} \cdot \text{ATP}}{\theta_{\text{tx}} + \text{ATP}}, \tag{S8}$$

where ω_{r} is an aggregate term accounting for maximal elongation rate, initiation rate and the length of the gene.

Transcription of *rtcBA* firstly requires the activation of RtcR. Since RtcR has been shown to form apparent hexamers before full activation [1], we describe RtcR activation via the established Monod-Wyman-Changeux (MWC) model [2]. In this case, six ligands cooperatively bind to each monomer in the RtcR hexamer:

$$p_R + 6r_t \rightleftharpoons p_R^*. \tag{S9}$$

Using MWC we can define a fraction of active RtcR (F_a) as follows,

$$F_a = \frac{(1+\alpha)^6}{L \cdot ((1 + c \cdot \alpha)^6) + (1+\alpha)^6}, \tag{S10}$$

with $\alpha = \frac{[\text{ligand}]}{K_R}$ the normalised concentration of ligand, $c = \frac{K_R}{K_T}$ the ratio of dissociation constants in R (inactive or 'relaxed') and T (active or 'tense') states and $L = \frac{[T_0]}{[R_0]}$ the allosteric constant, or the ratio of proteins in T and R states in the absence of any ligand. We choose L and c such that we can account for spontaneous activation of RtcR (see Table S1). Cooperativity is more marked when L is large and when c is small [2, 3]. We use a small value of c to ensure a high level of cooperativity, but we choose a smaller value of L to ensure an equilibrium in favour of the inactive R state, therefore a small fraction of

RtcR will be in the active state in the absence of ligand. To calculate the total amount of active RtcR (p_R^*) we multiply the fraction of active RtcR by the total amount of RtcR,

$$p_R^* = p_R \cdot F_a. \tag{S11}$$

Active RtcR (p_R^*) initiates transcription of *rtcBA* through the conversion of the closed holoenzyme complex (σ_c) to the open holoenzyme complex (σ_o) in a reaction dependent on ATP with an overall rate of v_{oc}. This is followed by the dissociation of the holoenzyme with reformation of σ_c at a rate of k_{diss} and subsequent initiation of transcription. Using mass action kinetics and assuming σ_o is in quasi-steady state we derive an equation for σ_o:

$$\sigma_o = \frac{p_R^* \cdot v_{\text{oc}}}{k_{\text{diss}}}, \tag{S12}$$

where

$$v_{\text{oc}} = \frac{V_{\text{max}} \cdot \text{ATP}}{K_m + \text{ATP}}, \tag{S13}$$

derived from partition analysis [4] from the overall reaction of σ_o formation:

$$p_R^* + \sigma_c \underset{k_2}{\overset{k_1}{\rightleftharpoons}} p_R^*\sigma_c \underset{k_4}{\overset{k_3 \cdot \text{ATP}}{\rightleftharpoons}} p_R^*\sigma_c\text{ATP} \overset{k_{\text{init}}}{\rightarrow} \sigma_o + p_R^*, \tag{S14}$$

where V_{max} and K_m are aggregate rate terms with values constrained from the literature shown in Table S1. Finally, the overall rate of transcription of *rtcBA* is

$$v_{\text{tx}}^{(\text{BA})} = \sigma_o \cdot \frac{\omega_{\text{ba}} \cdot \text{ATP}}{\theta_{\text{tx}} + \text{ATP}}, \tag{S15}$$

where ω_{ba} is an aggregate term considering elongation and the length of the gene. Compared to transcription of *rtcR*, initiation is considered by the formation of σ_o as a separate process.

S1.2 Rtc-regulated RNA repair

We model both the RtcA and RtcB reactions in the same way,

$$p_A + \text{ATP} \underset{k_{-a}}{\overset{k_a}{\rightleftharpoons}} p_A : \text{ATP} + r_d \underset{k_{-2}}{\overset{k_2}{\rightleftharpoons}} p_A : \text{ATP} : r_d \overset{k_{\text{tag}}}{\rightarrow} r_t + p_A, \tag{S16}$$

$$p_B + \text{ATP} \underset{k_{-b}}{\overset{k_b}{\rightleftharpoons}} p_B : \text{ATP} + r_t \underset{k_{-2}}{\overset{k_2}{\rightleftharpoons}} p_B : \text{ATP} : r_t \overset{k_{\text{rep}}}{\rightarrow} r_h + p_B. \tag{S17}$$

Using partition analysis and assuming ATP saturation, we derive the overall rate equations for tagging and repair as:

$$v_{\text{tag}} = \frac{p_A \cdot r_d \cdot k_{\text{tag}}}{r_d + K_a}, \tag{S18}$$

$$v_{\text{rep}} = \frac{p_B \cdot r_t \cdot k_{\text{rep}}}{r_t + K_b}, \tag{S19}$$

where K_a and K_b represent $\frac{k_{-2}+k_{\text{tag}}}{k_2}$ and $\frac{k_{-2}+k_{\text{rep}}}{k_2}$ respectively. However, K_a and K_b are known parameters so we use the corresponding values from the literature [5, 6].

Damage in the model is introduced by irreversible action on healthy RNAs,

$$r_h \overset{k_{\text{dam}}}{\rightarrow} r_d, \tag{S20}$$

with an overall rate of damage therefore represented by

$$v_{\text{dam}} = k_{\text{dam}} \cdot r_h. \tag{S21}$$

S1.3 Comparison of predictions between rRNA and tRNA repair

Despite the differences in the contribution of tRNAs and rRNAs to translation, we did not observe a marked difference in the results from performing the same model analyses. The tRNA model still displayed bistability (Figure S2A). The parameters ATP, λ, ω_{ba} and ω_{r} showed the same trends in the tRNA model as they did in the rRNA model (Figure S2B). We performed the same inhibition analysis on the tRNA model to see if there was a difference in the control of the Rtc system when tRNA affects translation elongation. We saw that inhibition of RtcA has virtually no effect in the regime of inhibition levels tested (Table S2) compared to inhibition of RtcB and RtcR where we saw a similar effect to that of the rRNA model (Figure S10). Therefore, under both hypotheses of Rtc-regulated repair of rRNA and tRNA, our analysis suggests that inhibition of RtcB or RtcR alongside treatment with an antibiotic to potentiate antibiotic effects and reduce the incidence of Rtc-induced resistance.

S1.4 Model extensions

S1.4.1 Inhibition of Rtc proteins

We model inhibition by reversible binding of an inhibitor (I) to the Rtc proteins,

$$p_x + I \underset{k_u^I}{\overset{k_b^I}{\rightleftharpoons}} p_x^I. \tag{S22}$$

This leads to the addition of an ODE for the new protein:inhibitor complex,

$$\dot{p}_x^I = k_b^I \cdot p_x \cdot I - k_u^I \cdot p_x^I - \lambda \cdot p_x^I, \tag{S23}$$

and altering the ODE for the Rtc protein,

$$\dot{p}_x = v_{\text{tl}}^{(\text{x})} - \lambda \cdot p_x - k_b^I \cdot p_x \cdot I + k_u^I \cdot p_x^I. \tag{S24}$$

This includes the addition of three model parameters, binding and unbinding (k_b^I, k_u^I) of the inhibitor (I) to the protein, where I is the concentration of inhibitor in μM. For our inhibition analysis, we varied the binding rate of inhibitor to achieve stronger or weaker inhibition (see Table S2).

S1.4.2 Dynamic dilution and influx rates

For ribosomal repair, here we consider a model extension to account for the relationship between both the growth rate and the influx rate, to dynamic levels of ribosomes. Using empirical growth relations described in [7], we outline two new equations:

$$\lambda = \lambda_c \cdot (r_{\text{max}} - r_h), \tag{S25}$$

$$k_{\text{in}} = k_{\text{in}}^{\text{max}} \cdot \frac{r_h}{1 + r_h}. \tag{S26}$$

Since we primarily study the model under conditions of translational inhibition due to the assumption that damage is inflicted by ribosome-targeting antibiotics, the growth rate is assumed negatively proportional to the healthy ribosomes (Eq. (S25)). This is in accordance with the second growth law presented in [7], $r = r_{\text{max}} - \frac{\lambda}{k_{\text{in}}}$. Since ribosomes catalyse their own synthesis, we assume the influx rate of ribosomes is positively correlated with dynamic ribosome levels, where we include a saturating term to avoid exponentially growing influx at high ribosomal concentrations (Eq. (S26)).

This model extension includes three new parameters, λ_c, $k_{\text{in}}^{\text{max}}$ and r_{max}, all of which can be found in Table S3 and replace the original λ and k_{in} parameters from the rRNA model when considering dynamic dilution and ribosome influx. Analysis of this model is shown in Figure S8 which is a repeated analysis carried out in Figure 3 from the main text. We note that the model with or without dynamic dilution and influx produces qualitatively the same steady-state response, particularly regarding bistability, suggesting that positive feedback, through the regulation of Rtc, appears sufficient to produce the bistable response without the need for growth-dependent feedback via dilution and influx.

S1.4.3 Direct repair via RtcB only

It is possible that some RNA damage may not produce 3' RNA ends but in fact produce 2',3'-cP ends [8]. In this scenario RtcB is able to directly repair 2',3'-cP ends, and RtcA is not required. We model this scenario by removing RtcA mRNA and protein species and removing damaged RNAs. Therefore, tagged RNAs are directly produced by damage at rate k_{dam}, and we no longer have a 'tagging' step in the repair. The scenario yields a modified equation for tagged RNAs

$$\dot{r}_t = r_h \cdot k_{\mathrm{dam}} - v_{\mathrm{rep}} - \lambda \cdot r_t. \qquad \text{(S27)}$$

Figure S1 shows a comparison of the dynamic responses when considering damage that directly yields 2'3'-cyclic phosphate ends, which can be repaired by RtcB without action by RtcA, and when considering damage that yields 3'-phospate RNA ends, which require the concerted action of RtcA and RtcB. Steady-state responses between the two model versions are largely equivalent, i.e. bistability in *rtc* expression and translational capacity with largely the same steady-state values except for minor deviations in a small range of damage rates (Figure S1 top). There are also minor differences between the two scenarios in their transient behaviour, as steady states are approached more rapidly when damage directly yields the ligand that induces rtc expression (Figure S1 bottom).

S2 Supporting Methods

S2.1 Quantification of *rtc* expression with qPCR

The data shown in Table S4 was obtained using real-time quantitative PCR (RT-qPCR). *E.coli* MG1655 was grown overnight on LB and transferred to a liquid culture where samples were incubated at 37°C on a shaker for 4, 6, 8, 10, 12 and 24 hours. At the respective time points, total RNA was extracted using the Qiagen RNeasy Protect Bacterial mini kit. The samples were then treated with DNase I (Promega) and reverse transcription was carried out using the SuperScript III Reverse Transcriptase. RT-qPCR was performed with the OneStepPlus Real-Time qPCR System from Applied Biosystems. Power SYBR Green PCR Master Mix also from Applied Biosystems was used. Table S4 shows expression levels at 10 hrs when they reached maximal levels.

Table S1: Model parameters.

Parameter	Description	Value	Unit	Ref
L	Allosteric constant	50	Unitless	[2]
c	Ratio of dissociation constants	0.01	Unitless	[2]
θ_{tx}	Transcription threshold	160.01 §	μM	[9]
θ_{tl}	Translation threshold	255.73 §	μM	[9]
k_{tag}	Tagging rate of RtcA	9780 ± 840	min^{-1}	[10]
k_{rep}	Repair rate of RtcB	15.67 ± 1.25	min^{-1}	[11]
V_{max}	Maximal rate of open complex formation	39.51 ± 4.62	min^{-1}	[12, 13]
K_m	Michaelis constant for open complex formation	250 ± 50	μM	[12, 13]
d_m	mRNA degradation rate	0.2	min^{-1}	[14]
ATP	Intracellular ATP concentration	3000	μM	[15–17]
K_a	Michaelis constant of RtcA for ATP	20	μM	[5]
K_b	Michaelis constant of RtcB for ATP	16	μM	[6]
n_a	Length of RtcA	338	aa	[18]
n_b	Length of RtcB	408	aa	[18]
n_r	Length of RtcR	532 †	aa	[18]
γ_{max}	Maximal translation rate	1260	$\mathrm{aa} \cdot \mathrm{min}^{-1}$	[9]
λ	Growth rate	0.014	min^{-1}	[7]
k_{deg}	Degradation rate of damaged RNAs	1	min^{-1}	‡
ω_{ba}	Maximal transcription rate of *RtcBA*	$5 \cdot 10^{-5}$	min^{-1}	‡
ω_{r}	Maximal transcription rate of *RtcR*	$1 \cdot 10^{-6}$	$\mu\mathrm{M} \cdot \mathrm{min}^{-1}$	‡
k_{dam}	Damage rate	0-0.8	min^{-1}	‡
k_{diss}	Holoenzyme dissociation	0.006	min^{-1}	[19]
k_c	Ribosome-mRNA binding constant	0.6	$\mu\mathrm{M}^{-1}$	[9]
	**Parameters specific to ribosome repair:**			
K_R	Affinity of active state for the ligand	0.125	μM	[20]
$k_{\mathrm{in}}^{\mathrm{rRNA}}$	Influx of healthy rRNA	0.255	$\mu\mathrm{M} \cdot \mathrm{min}^{-1}$	‡
	**Parameters specific to tRNA repair:**			
R	Concentration of ribosomes	35	μM	[21]
θ_{t}	Threshold for tRNA	100	μM	[22]
$k_{\mathrm{in}}^{\mathrm{tRNA}}$	Influx of healthy tRNA	2.55	$\mu\mathrm{M} \cdot \mathrm{min}^{-1}$	‡
K_R	Affinity of active state for the ligand	1.5	μM	*

§ Values have been scaled to account for effective resource levels, scaling factor (ζ) from [23].
† In the model we use six times this parameter value to account for the inclusion of the RtcR hexamer.
* Adjusted from ribosome repair to account for a greater number of tRNAs to rRNAs in a cell.
‡ Parameter values chosen as explained in section S2.2.

Table S2: Parameters for Rtc inhibition

Parameter	Description	Value	Unit	Ref
k_b^I	Rate of inhibitor binding (rRNA repair)	0.3, 0.5, 1	min^{-1}	‡
k_b^I	Rate of inhibitor binding (tRNA repair)	0.05, 0.1, 0.2	min^{-1}	‡
k_u^I	Rate of inhibitor unbinding	0.0025	min^{-1}	‡
I	Concentration of inhibitor	0.1	μM	‡

‡ Parameter values chosen as explained in section S2.2.

S2.2 Parameterisation

Degradation of damaged RNA (k_{deg}) was chosen to be faster than the rate of mRNA degradation (d_m) as the reactive 3' ends of damaged RNAs are likely less stable than mRNAs. The two maximal rates of transcription (ω_{ba} and ω_{r}) were chosen based on model steady-state values in non-inducing conditions ($k_{\mathrm{dam}} = 0$) closely matching that of mRNA concentrations determined by qPCR (Table S4). Likewise,

Table S3: Parameters for dynamic dilution & ribosome influx rates

Parameter	Description	Value	Unit	Ref
λ_c	Growth rate constant	0.00068	min^{-1}	‡
r_{max}	Maximal healthy ribosomes	38.8	μM	‡
$k_{\text{in}}^{\text{max}}$	Influx constant	0.268	$\mu\text{M} \cdot \text{min}^{-1}$	‡

‡ Parameter values chosen as explained in section S2.2.

Table S4: qPCR data used for tuning maximal transcription rates. Data have been acquired as described in S2.1 and were used to tune the ω parameters found in Table S1.

mRNA	Concentration (μM)
rtcA	5.31x10^{-6}
rtcB	2.66x10^{-5}
rtcR	5.31x10^{-7}

the influx of healthy RNAs ($k_{\text{in}}^{\text{rRNA}}$, $k_{\text{in}}^{\text{tRNA}}$) was chosen so that model steady-state concentrations of protein matched those of RtcA and RtcB protein concentrations found on the EcoCyc database and to be approximately 1000 times bigger than the concentration of steady-state mRNA [18, 24]. We varied the damage parameter (k_{dam}) across a wide range, always starting from zero, representing a drug-free situation, so as not to limit the range of damage conditions that different drug concentrations may cause. We then narrowed this range down to regions where we observed interesting activity for both the ribosome and tRNA repair model. Parameters for inclusion of an inhibitor into the model were chosen so that the binding rate was faster than the unbinding rate in all conditions tested. Finally, the concentration of inhibitor is very dependent on the specific drug, so this was chosen to see a response with inhibitor addition to the model, separately for both the ribosome and tRNA repair model. Additional parameters for the dynamic growth rate and influx model (Table S3) were chosen to see comparable steady-state concentrations to the main rRNA and tRNA models.

S2.3 Sensitivity analysis

We performed a sensitivity analysis of model predictions regarding bistability. We sampled parameters from normal distributions with mean values found in the literature, and for parameters where standard deviations were reported (see Table S1), we doubled the standard deviations for more exhaustive exploration of the parameter space. In cases where standard deviations were not found in the literature, we sampled 20% either side of the mean values. In total, we perturbed 15 parameters as seen in Figures S6 and S7. We sampled each parameter independently and considered a total of five million samples of parameter combinations. For each sample, we performed a stability analysis to determine if the new set of parameters gave rise to bistability. We found that when perturbing the 15 parameters within the ranges specified from literature uncertainties, bistability was displayed in 95.55% and 87.2% of the five million parameter combinations used for the rRNA and tRNA models respectively, suggesting that bistability in the Rtc system is robust. For the model of ribosome repair, we observed λ, k_{in} and the rate of ribosome-mRNA binding, k_c, to have the greatest effect on stability. These parameters influence the overall species concentrations, the concentration of ribosomes and the translation rate. Extremes of λ and values of k_c and k_{in} being too low will lead to a reduced number of Rtc proteins and ribosomes and therefore loss of bistability. For the model of tRNA repair, we observed a similar response, however the influx of tRNAs had a weaker effect compared to the model of ribosome repair, potentially because there are so many more tRNAs contributing to the translation rate than ribosomes.

S2.4 RNA FISH probe design

The Stellaris® Probe Designer was used to design probes complementary to the *rtcB* gene sequence (Table S6). The design parameters were as follows. Masking level: 2, oligo length: 20, minimum spacing length: 20.

Table S5: Probe sequences of RNA probes designed complementary to the *rtcB* gene sequence.

**Probe**	**Sequence (5' to 3')**	**Probe**	**Sequence (5' to 3')**
rtcB_1	gcattttcagtggtcagtaa	rtcB_24	caacgtctcaagcgtttcct
rtcB_2	tttggtccacatttttaccg	rtcB_25	aagtacgccagatcacgcga
rtcB_3	aataagttgctgacgcgcat	rtcB_26	aaagtattccgtaccttcca
rtcB_4	taaacggcatcttcgccgta	rtcB_27	ccacggctttcaggtaatca
rtcB_5	cattaccgcaatatgtttga	rtcB_28	gcggttaaggctggcaaaaa
rtcB_6	ctttacccaggtgtacatca	rtcB_29	ttaccacgttttccatcatc
rtcB_7	atcacgctaccaatggtgga	rtcB_30	tgaccgttttctgcgtaatg
rtcB_8	gaataatcgcccctttggtc	rtcB_31	tggtgacagttgatctcttc
rtcB_9	ttcattccacagccaatatc	rtcB_32	tgctgttctttttgcacata
rtcB_10	aggttttcaggcaggtcttc	rtcB_33	cacgtagatctcttcaccaa
rtcB_11	gtttcaatcgcctgacgcag	rtcB_34	atattgaccagcacgcgcag
rtcB_12	ttatcacgtttacaacggcc	rtcB_35	ccatcgaaccgggaataatt
rtcB_13	cgttaacaggtggattttcc	rtcB_36	ccacggacgataaagctttt
rtcB_14	ttcaagctcagcccatttag	rtcB_37	cagaacgactcttcatttcc
rtcB_15	tgcgttaaccactgataacc	rtcB_38	ctttagttcggctcattacc
rtcB_16	ggtattcaggaaacggggat	rtcB_39	tccacgctgaacagtttttt
rtcB_17	ttcccaggtgtttatagtta	rtcB_40	cgcggtggcacgaatttgat
rtcB_18	taaagtggttaccggttccc	rtcB_41	catctttacggcattccaca
rtcB_19	gactcatcaaggcagatttc	rtcB_42	atcgggatttcgtcgatcac
rtcB_20	cagcataatccacacctggt	rtcB_43	catcaccgcatcaatatctt
rtcB_21	caattccgcgtgaaccggag	rtcB_44	gggtatagataacttccacc
rtcB_22	taaagtaagtcccgatggcg	rtcB_45	tatccttttacgcacaccac
rtcB_23	atctctttttgtgccagatc		

S2.5 Materials

Table S6: Materials detailing manufacturer and catalogue number.

**Compound**	**Manufacturer**	**Catalogue number**
4-methylumbelliferyl-D-galactopyranoside	Sigma Aldrich	M9766
Agarose	Thermo Fisher Scientific	10766834
Aspartate	Sigma Aldrich	A6558
ATP	Thermo Fisher Scientific	R0441
β-mercaptoethanol	Sigma Aldrich	444203
Chloramphenicol	Sigma Aldrich	C0378
DAPI	Thermo Fisher Scientific	D1306
DEPC-treated water	Invitrogen	AM9922
Dextran sulfate sodium salt	Sigma-Aldrich	42867-25G
E. coli tRNA	Sigma-Aldrich	R1753-500UN
Ethanol Honeywell	Riedel-de Haën	603-002-00-03
Formaldehyde	Sigma Aldrich	47608
Formamide	Thermo Fisher Scientific	10602882
Fructose-1,6,-Bisphosphate (FBP)	Sigma Aldrich	F6803
Gentamicin	Sigma Aldrich	G1272
Glucose	Sigma Aldrich	49139
Glutamate	Sigma Aldrich	G1501
Glutamine	Sigma Aldrich	49419
Glutathione	Sigma Aldrich	G4251
GTP	Thermo Fisher Scientific	R0461
HEPES	Sigma Aldrich	H23830
IPTG	Sigma Aldrich	I1284
LB broth	Sigma Aldrich	L3522
Lignin	Sigma Aldrich	370959
Lipid extract	Avanti Research	100600C
NAD+	Sigma Aldrich	N0632
PBS	Invitrogen	AM9624
Ribonucleoside-vanadyl complex	New England Biolabs	S1402S
RNA FISH probes	LGC Biosearch Technology	
Saline-sodium citrate (SSC)	Merck Millipore	567780
Tetracycline	Sigma Aldrich	T7660
UTP	Thermo Fisher Scientific	R0471
Valine	Sigma Aldrich	94619

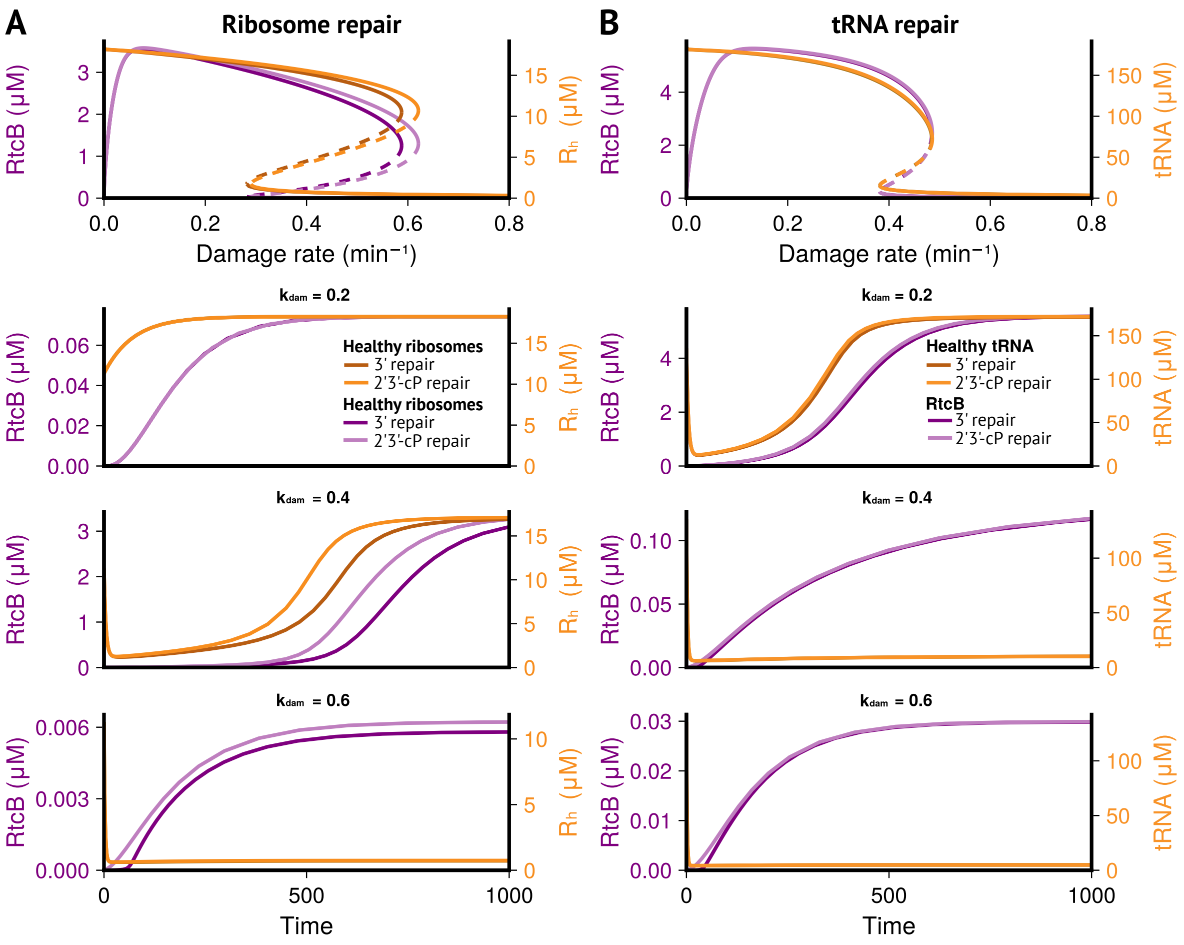

Figure S1: **Direct repair via RtcB only.** Comparison between scenarios where damage yields 2'3'-cP RNA ends (direct repair by RtcB only) and 3'-P termini (concerted repair by RtcBA as considered in the main models) for ribosome (rRNA) repair (A) and tRNA repair (B). RtcB (purple) is displayed on the left y-axis and healthy RNA (orange) is displayed on the right y-axis. Darker shades of purple or orange represent predictions for repair of 3' ends and lighter colours represent predictions for direct repair of 2'3'-cP ends via RtcB only. (Top row) Bifurcation diagrams display minor differences in steady-state values between 3' repair and the 2',3'-cP repair for rRNA and no difference for tRNA. (Bottom panels) Time-resolved trajectories at exemplar damage rates demonstrate that steady states are generally reached more rapidly in the case of 2'3'-cP repair. Differences are minimal in the tRNA model at all damage rates.

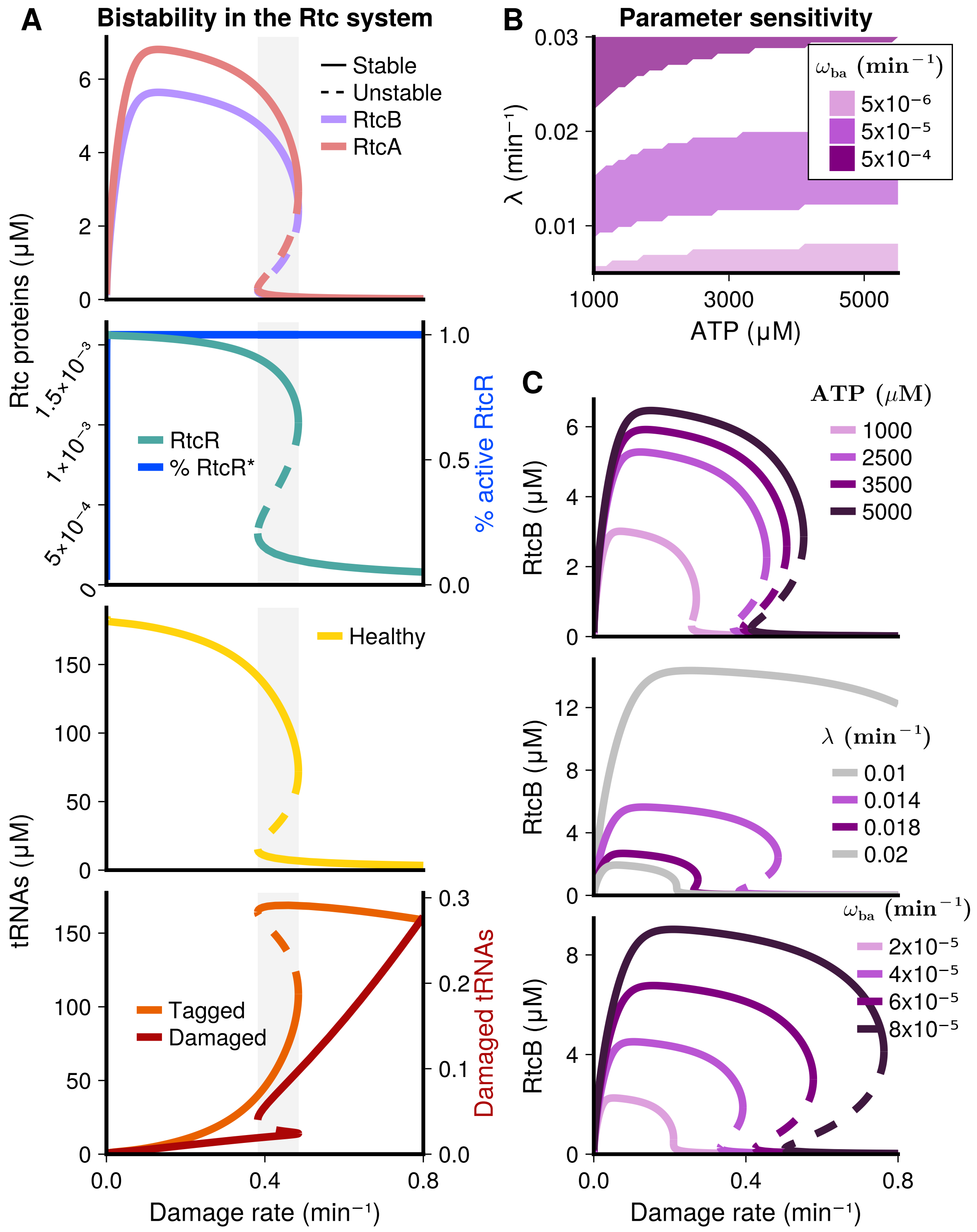

Figure S2: **Steady-state responses of the tRNA model.** (A) Stability analysis: Bifurcation diagrams for the Rtc proteins (top) and tRNA species (bottom) show a range of damage rates (grey region) where two stable cell states (solid lines) and one unstable state (dashed lines) coexist. (B) Like the ribosome-repair model, the model of tRNA repair displays bistability across a range of parameter conditions. Purple regions represent where combinations of ATP and dilution rate display bistability across the range of damage rates tested (0 to 0.8 min^{-1}). The shades of purple indicate how this region is affected by the inducibility of *rtcBA* through ω_{ba}. Higher inducibility allows bistability to occur at a wider range of higher dilution rates. (C) ATP (top), dilution rate (middle) and ω_{ba} (bottom) all affect the area of bistability and *rtc* expression.

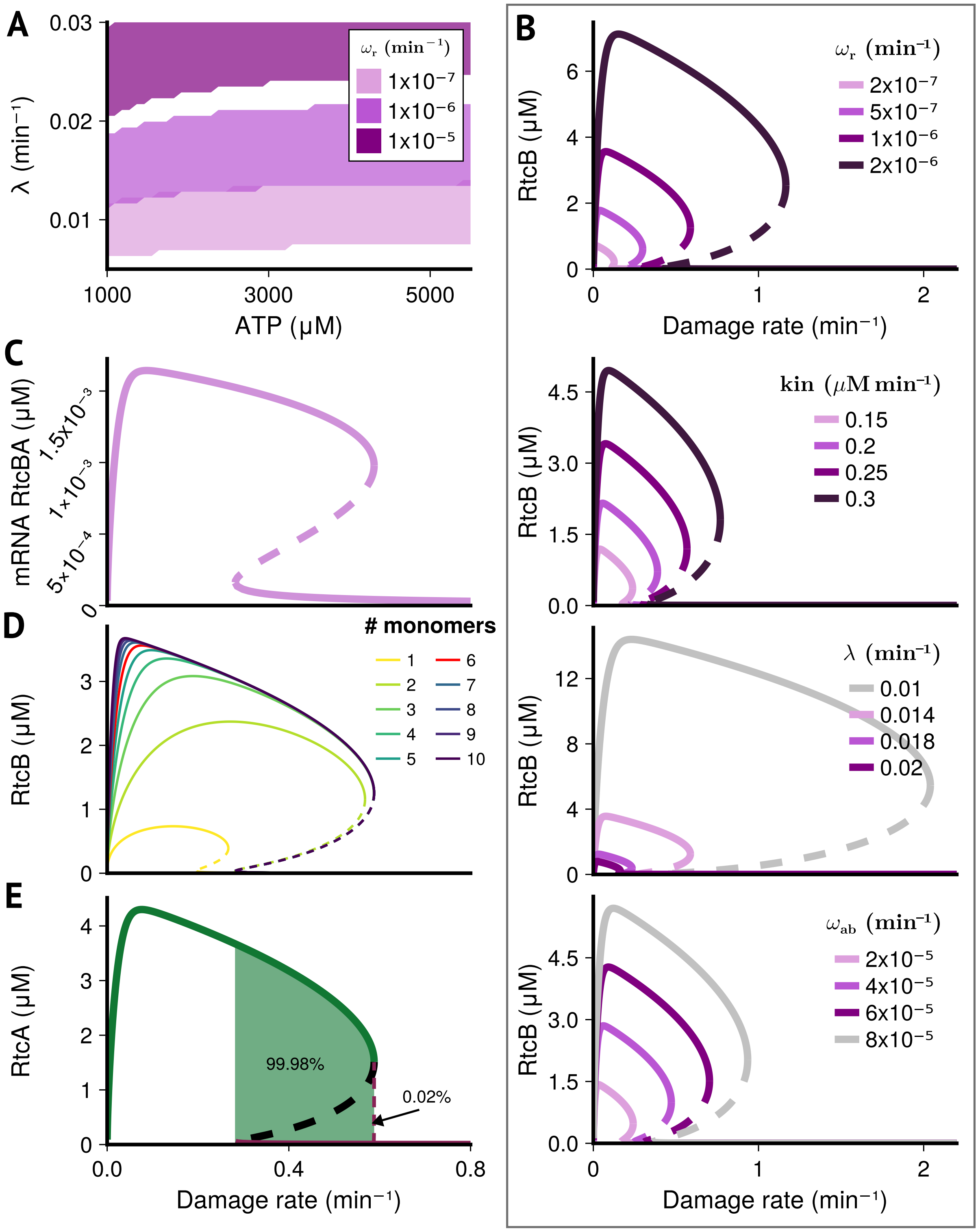

Figure S3: **Additional analysis of ribosome repair.** (A) Shown is the effect of *rtc* inducibility controlled by ω_r, which affects the bistable response in the same way as ω_{ba} seen in Figure 3 in the main text. (B) Bifurcation diagrams for different ω_{ba} (bottom) and λ (bottom middle) show that the grey lines shown in Figure 3C are bistable responses when considering a larger range of damage conditions. We also show here parameter sensitivity for the additional parameters ω_r (top) and the influx rate of healthy ribosomes k_{in} (top middle) where concentration of Rtc protein and size of the bistable region increase upon increasing parameter values. (C) The model predicts that mRNA concentrations of RtcBA also display a bistable response to damage. (D) Sensitivity of predictions with respect to the number of ligands that bind RtcR for activation. Cooperativity is not required for a bistable response given that bistability is maintained even when only one ligand binds RtcR. (E) Perturbing initial concentrations of RtcA (equivalent to the analysis shown in Figure 5A for RtcB and RtcR) is predicted to be effective in switching to the 'off' state only for perturbations in the very upper section of the bistable region.

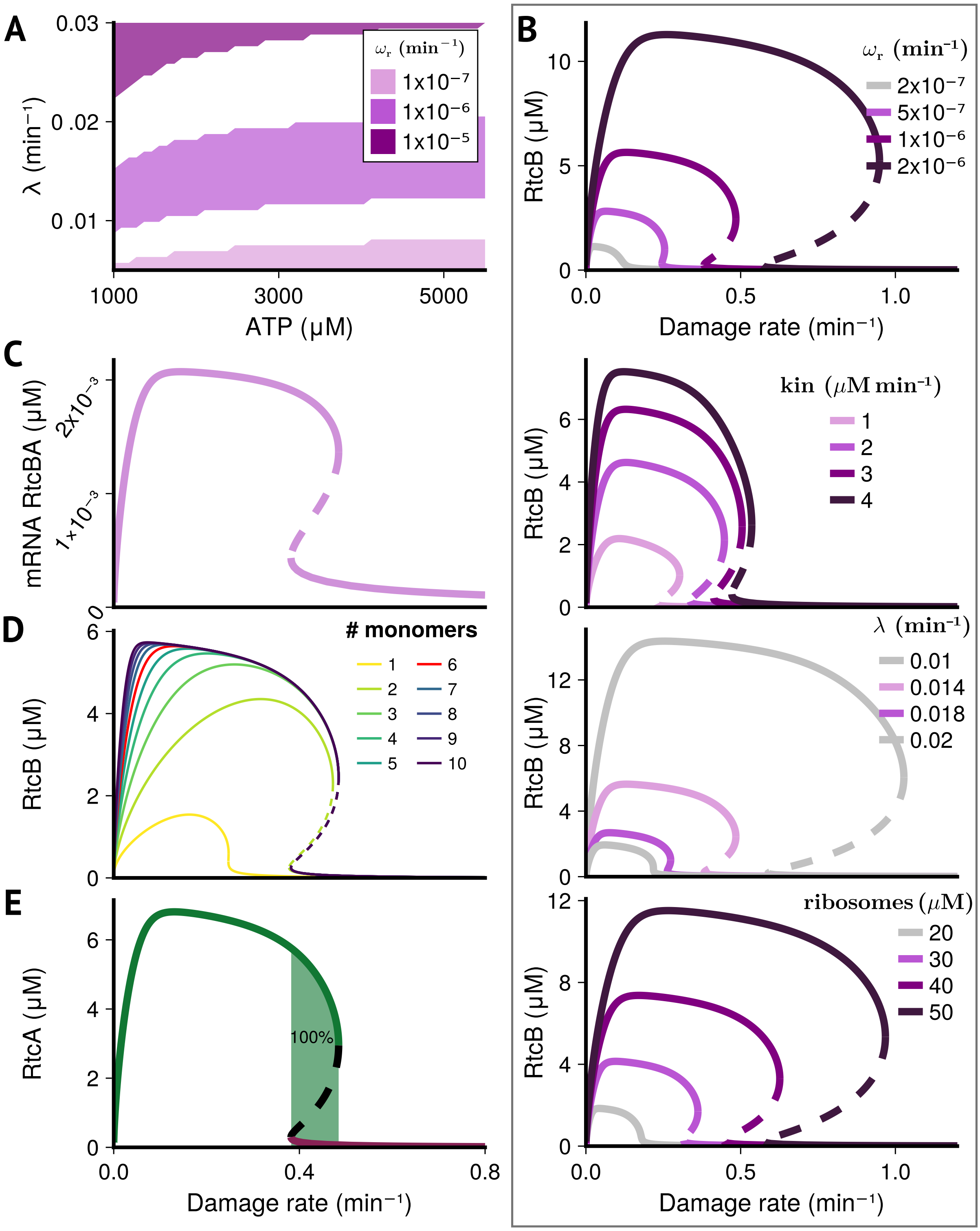

Figure S4: **Additional analysis of tRNA repair.** (A) Shown is the effect of *rtc* inducibility controlled by ω_r, which affects the bistable response in the same way as ω_{ba} seen in Fig S2B. (B) The bifurcation diagram for different λ (bottom middle) shows that the grey lines shown in Figure S2C are a bistable response when considered for a larger range of damage conditions. We also show here parameter sensitivity for the additional parameters ω_r (top) and k_{in} (top middle) and ribosomes (bottom) where concentration of Rtc protein and size of the bistable region increase upon increasing parameter value. (C) The model shows that mRNA concentrations of RtcBA also show a bistable response to damage. (D) Sensitivity of predictions with respect to the number of ligands that bind RtcR for activation. Cooperativity is not required for a bistable response given that bistability is maintained even when only one ligand binds RtcR. (E) Perturbing initial concentrations of RtcA (equivalent to the analysis shown in Figure S10A) is predicted to be insufficient for switching to the 'off' state.

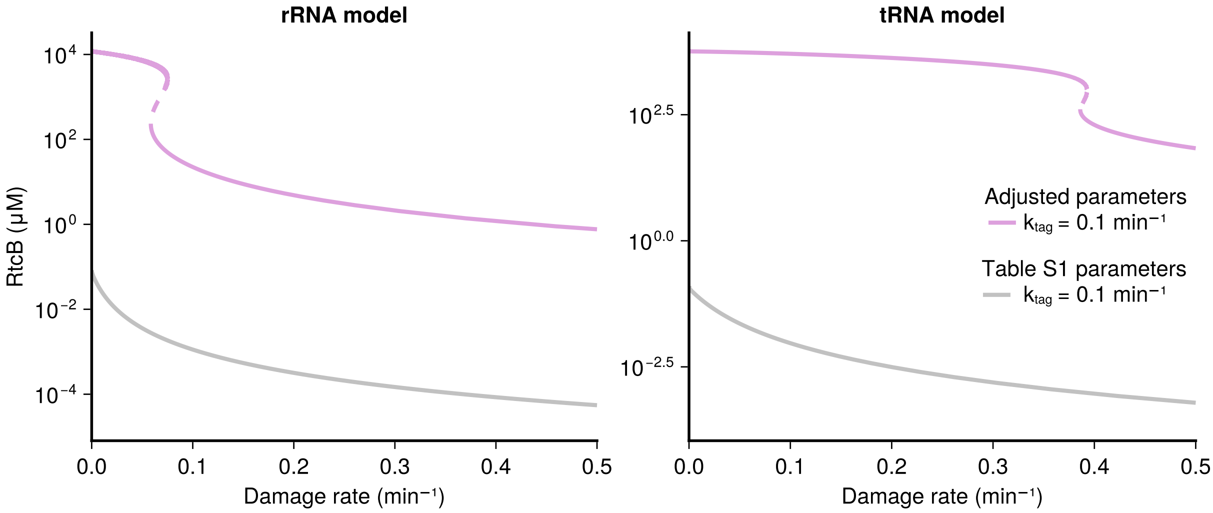

Figure S5: **The cyclisation of 2'-phosphate RNA termini by RtcA gives a bistable response in extreme parameter regimes.** For both the rRNA and tRNA models, the mechanism of repair of a 2'-phosphate termini via RtcA (k_{tag}: 0.1 min^{-1}), leads to a monostable response in the parameter regime presented in Table S1 (grey curves) and a bistable response in an adjusted parameter regime (purple curves). The adjusted parameters are as follows: λ : 0.0038 min^{-1}, ω_{ba} : $8 \cdot 10^{-4}$ min^{-1}, ω_r : $2 \cdot 10^{-5}$ μM ·min^{-1}. rRNA model specific parameters: $k_{\text{in}}^{\text{rRNA}}$: 0.4 μM ·min^{-1}, tRNA model specific parameters: $k_{\text{in}}^{\text{tRNA}}$: 4 μM ·min^{-1}, R : 80 μM.

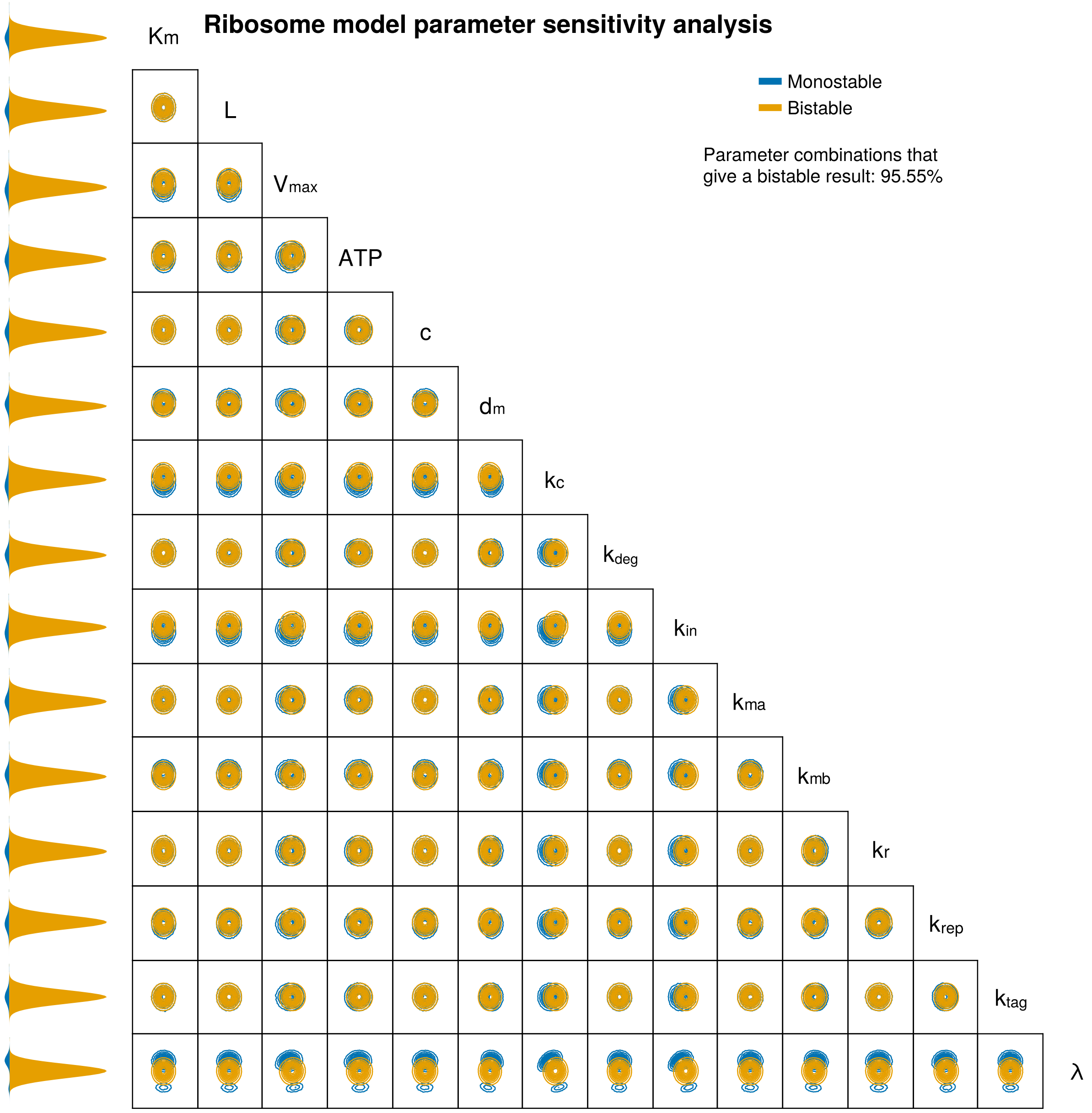

Figure S6: **Sensitivity analysis of bistability in the rRNA-repair model.** Contour plots show the 2D parameter distributions for both bistable (orange) and monostable (blue) distributions with each row and column corresponding to the parameters labelled along the diagonal. Violin plots for each parameter in each row show the absolute frequencies of bistable and monostable responses. Very few parameter combinations gave rise to a monostable response for the rRNA model, hence the small blue distributions seen on the left of these plots.

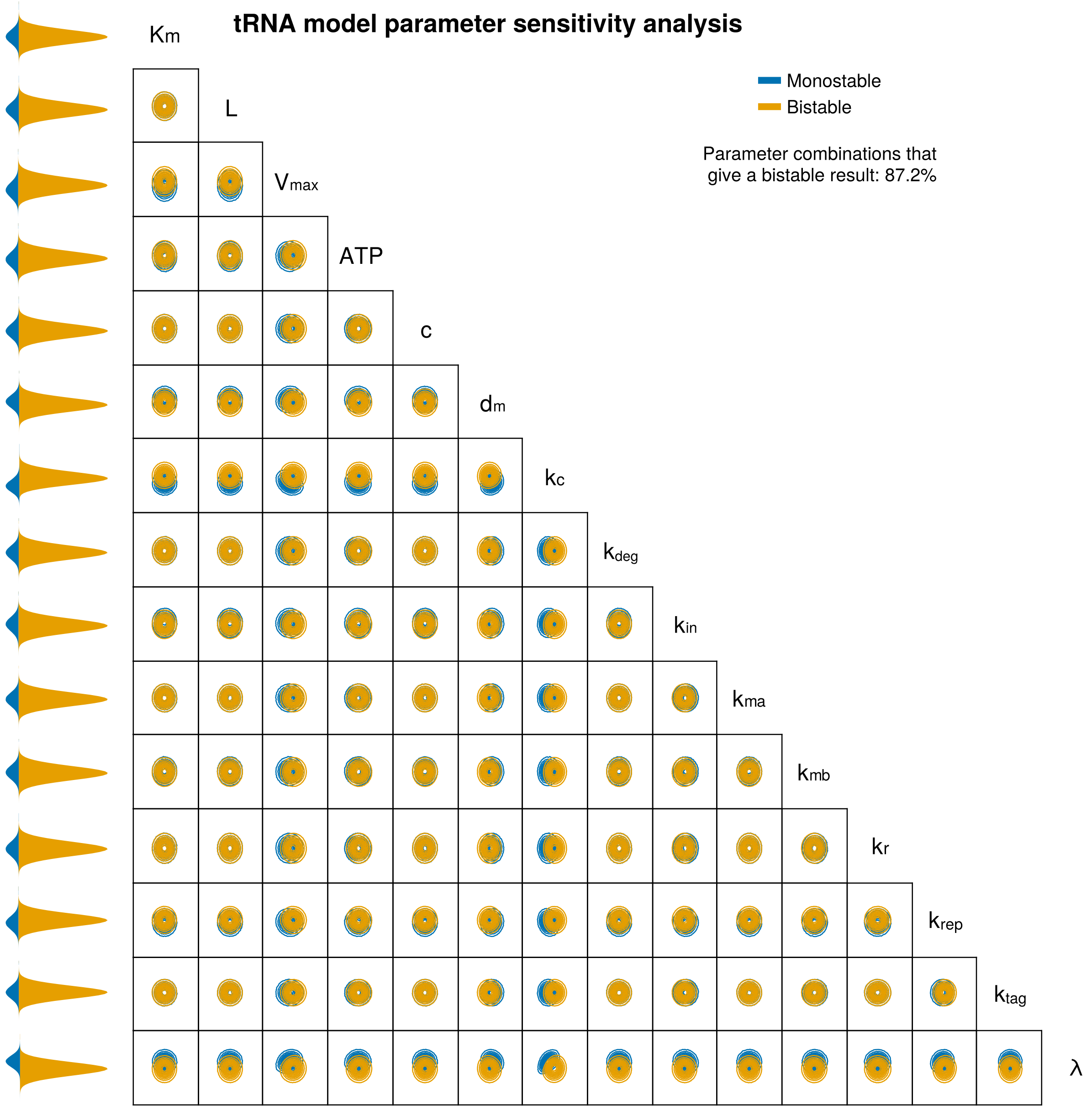

Figure S7: **Sensitivity analysis of bistability in the tRNA-repair model.** Contour plots show the 2D parameter distributions for both bistable (orange) and monostable (blue) distributions with each row and column corresponding to the parameters labelled along the diagonal. Violin plots for each parameter in each row show the absolute frequencies of bistable and monostable responses.

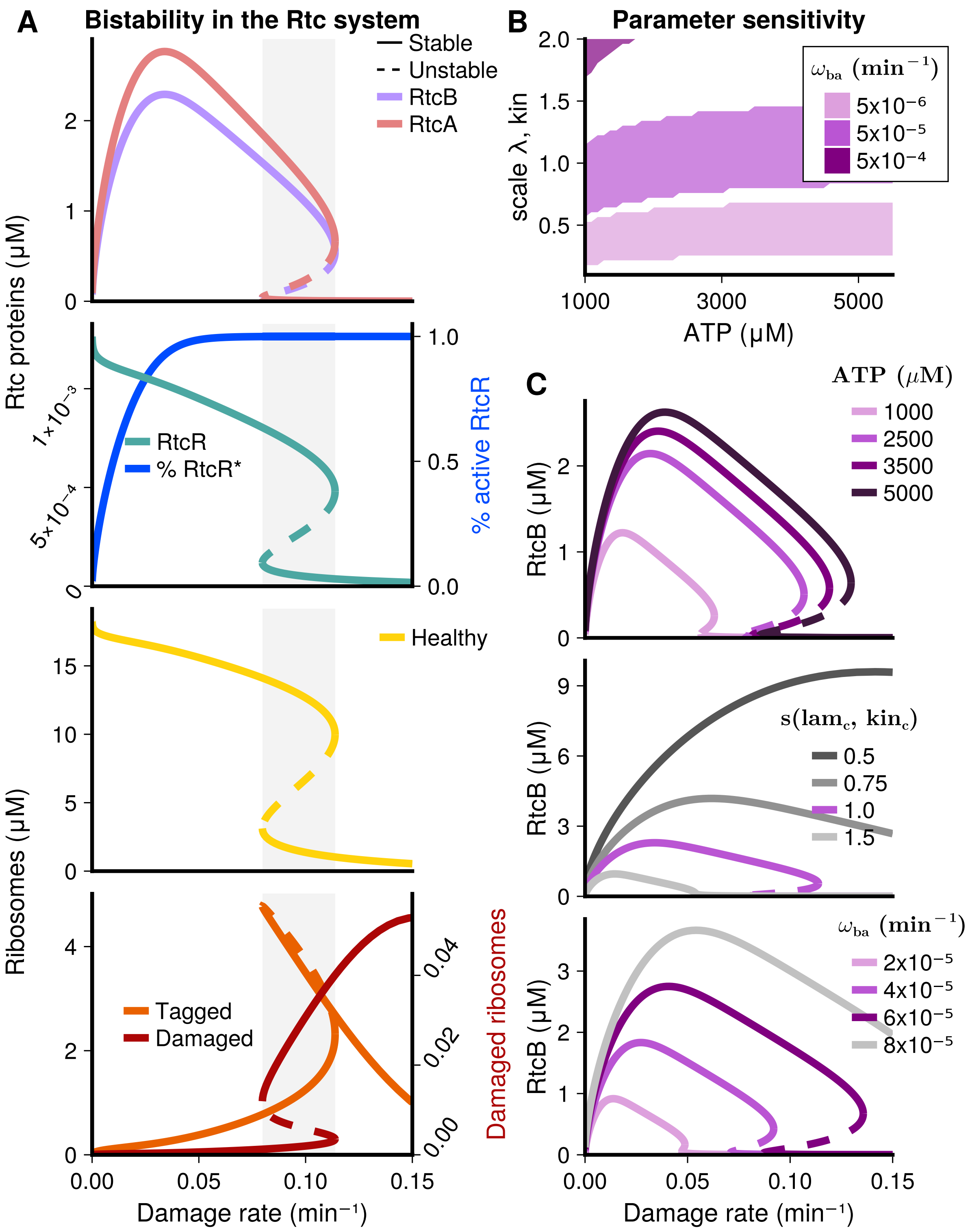

Figure S8: **Steady-state responses when considering ribosome repair with dynamic dilution and influx rates.** (A) Stability analysis: Bifurcation diagrams for the Rtc proteins (top) and rRNA species (bottom) show a range of damage rates (grey region) where two stable cell states (solid lines) and one unstable state (dashed lines) coexist. (B) Similar to the main model (with constant dilution and influx), this model displays bistability across a range of parameter conditions. Purple regions show where combinations of ATP and a scaling factor for the linking constants (λ_c and $k_{\mathrm{in}}^{\mathrm{max}}$) display bistability across the range of damage rates tested (0 to 0.8 min^{-1}). We multiplied both λ_c and $k_{\mathrm{in}}^{\mathrm{max}}$ by the scaling factor to sweep these parameters together as they are inherently linked. The shades of purple indicate how this region is affected by the inducibility of *rtcBA* through ω_{ba}. (C) ATP (top), scaling factor for the linking constants (middle) and ω_{ba} (bottom) all affect the area of bistability and Rtc expression.

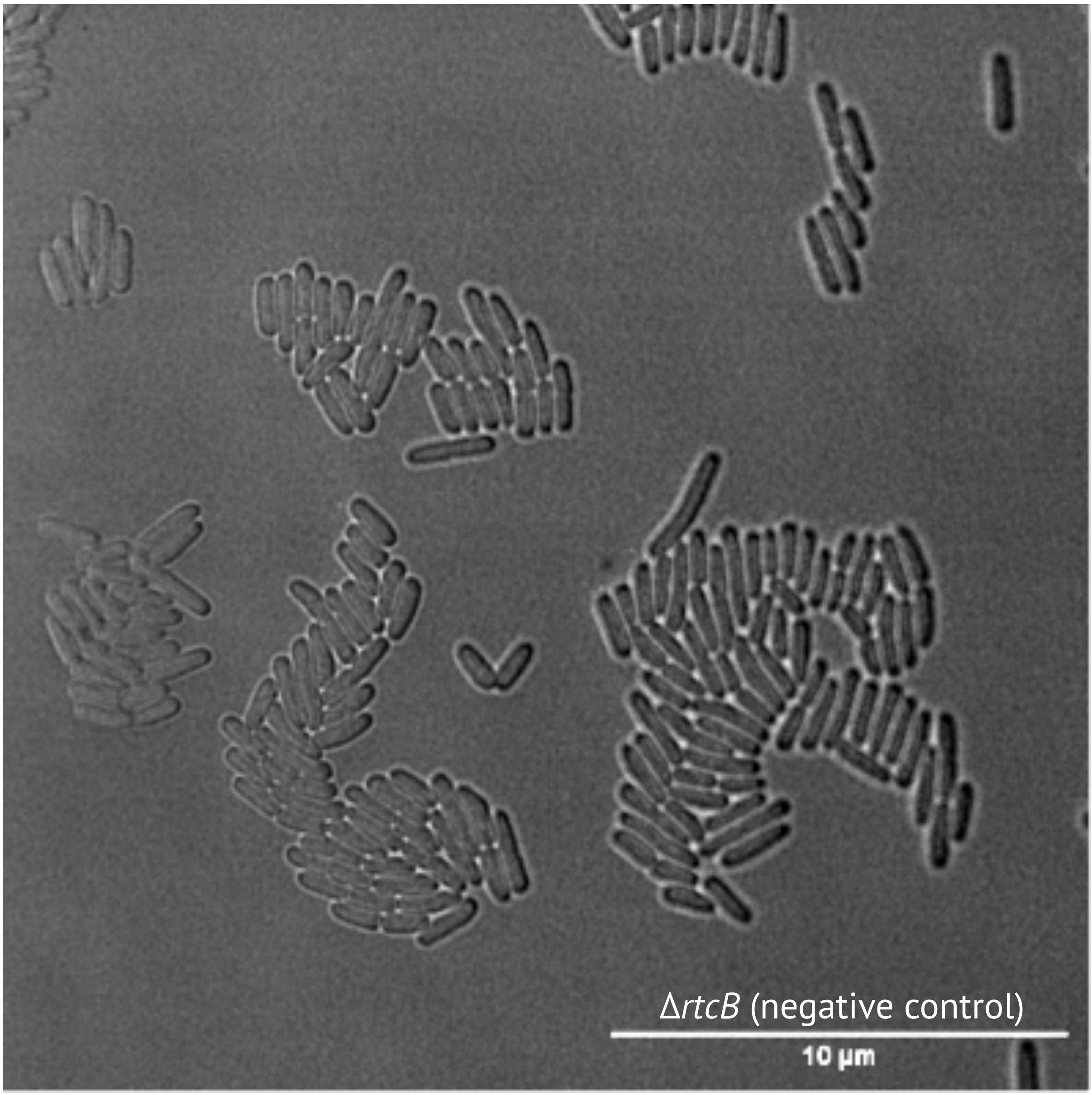

Figure S9: **Negative control for the smFISH experiment.** Overlayed brightfield and fluorescence microscopy images showing no fluorescence spots or presence of *rtcB* mRNA.

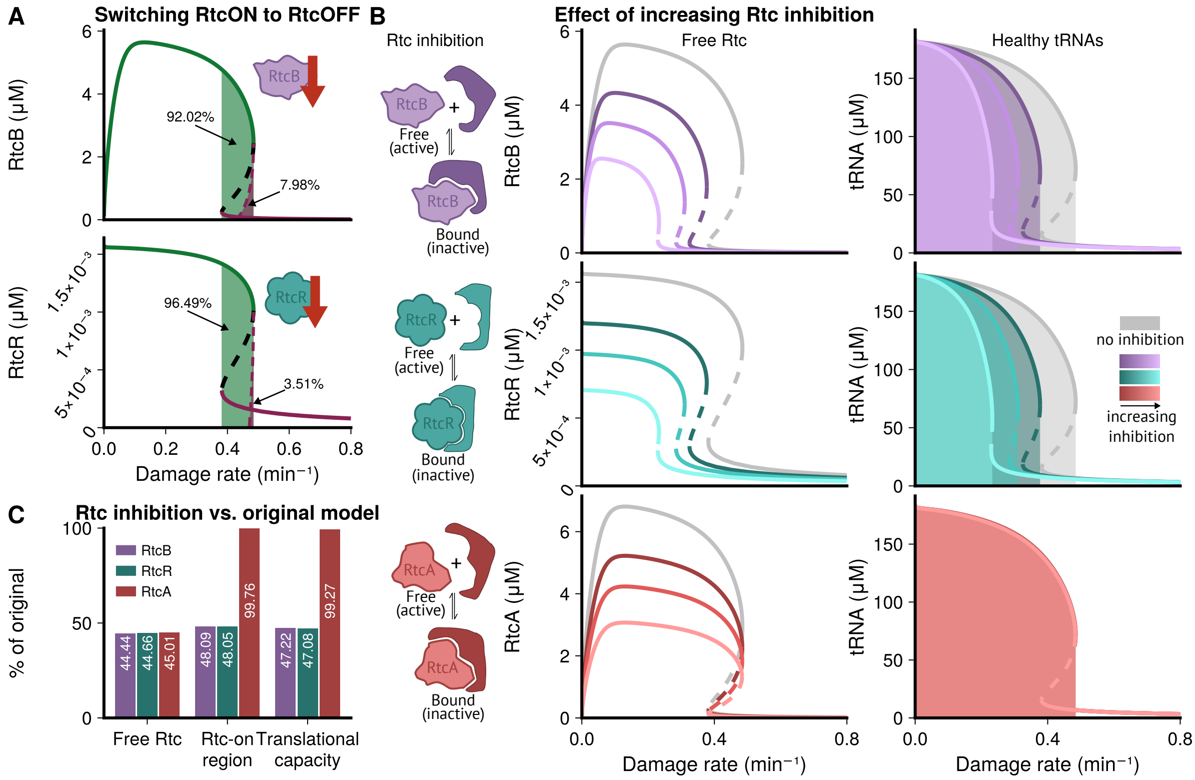

Figure S10: **Inhibiting Rtc proteins is predicted to reduce levels of resistance (tRNA repair).** (A) Perturbing the initial conditions of one molecular species at a time shows that decreasing initial conditions of RtcB (top) and RtcR (bottom) from their 'on' steady-state values can 'switch off' Rtc expression. Green and purple regions represent initial values where no switch is possible and values that cause a switch respectively. Switching is only possible at higher damage rates and RtcB is able to switch for more values at this top end than RtcR. This line is below the unstable steady state because we only perturb one initial condition at a time. (B) Inhibiting Rtc proteins decreases predicted concentrations of free Rtc (left panes). Inhibiting RtcB (top row) or RtcR (middle) reduces the range of damage conditions where Rtc is expressed and resistance to damage is possible. Inhibiting RtcA (bottom) decreases the concentration of free protein but hardly changes the range of damage rates where bistability is present. Healthy tRNA concentrations (right panes) decrease for RtcB and RtcR inhibition, but not for RtcA inhibition. (C) Summary of Rtc inhibition comparing the effects of the strongest level of inhibition of either RtcB, RtcR or RtcA to that of no inhibition.

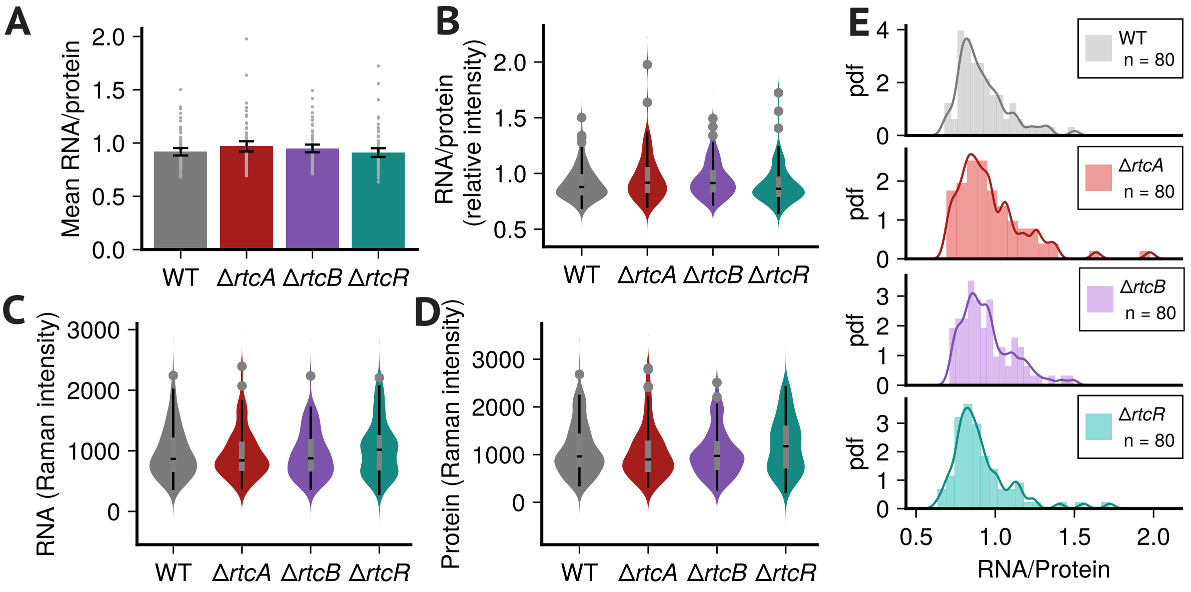

Figure S11: **Individual gene knockouts of the *rtc* genes do not impact ribosome levels in untreated cells.** (A) Mean RNA/protein ratios are not significantly different between WT ($n = 80$) and any *rtc* knockout strain ($n = 80$ for all strains) when performing a two-sided t-test ($\Delta rtcA$ t-statistic: -1.68, degrees of freedom (df): 144, p-value: 0.095, $\Delta rtcB$ t-statistic: -1.22, df: 158, p-value: 0.22, $\Delta rtcR$ t-statistic: 0.28, df: 154, p-value: 0.78). Error bars represent 95% confidence intervals. (B) Distributions of RNA/protein ratios. (C) Distributions of RNA and (D) protein concentrations as measured by Raman intensity. In untreated conditions all distributions of knockout strains were not significantly different to the WT when performing a one-sided Mann Whitney U test (p-values: RNA/protein $\Delta rtcA$: 0.09, $\Delta rtcB$: 0.09, $\Delta rtcR$: 0.24, total RNA: $\Delta rtcA$: 0.41, $\Delta rtcB$: 0.35, $\Delta rtcR$: 0.20, total protein $\Delta rtcA$: 0.77, $\Delta rtcB$: 0.74, $\Delta rtcR$: 0.84). (B)-(D) show box plots highlighting the median, interquartile range (IQR), and whiskers spanning the third and first quartiles $\pm$ 1.5$\times$IQR. (E) Histograms for each strain are shown with a kernel density estimate overlayed (solid lines). Each distribution displays one mode at the same RNA/protein ratio, signifying no difference to the WT. Source data are provided in a source data folder.

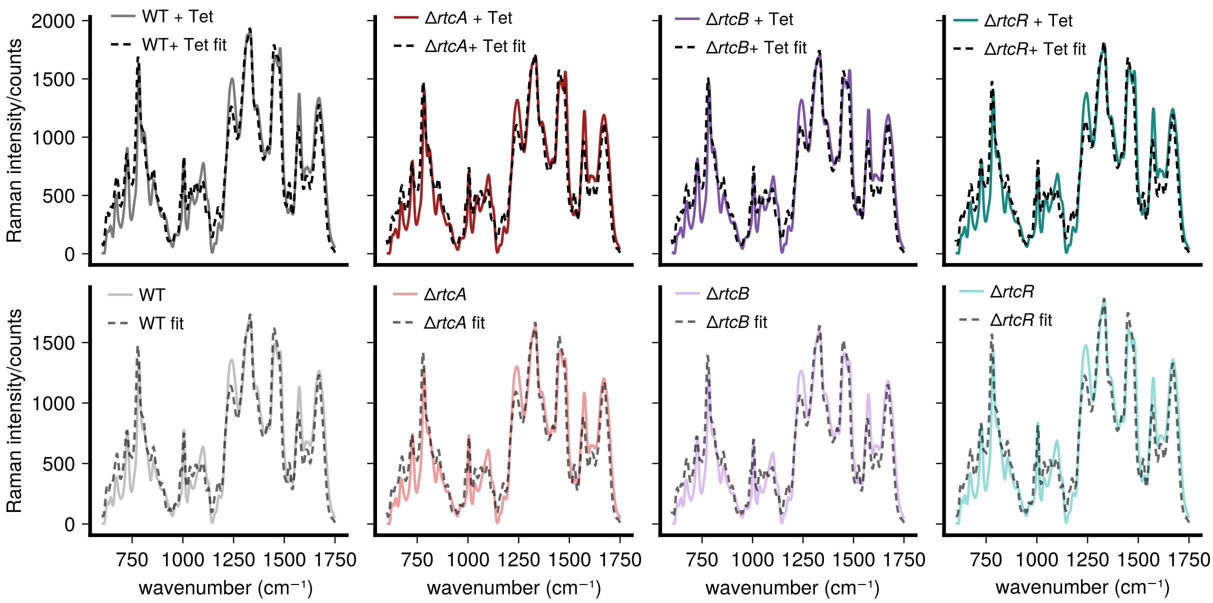

Figure S12: **Measured and fitted Raman spectra.** (Top) Average Raman spectra for the tetracycline-treated cells with associated fit. (Bottom) Average Raman spectra for the untreated cells with their corresponding fits. Cellular Raman spectra were decomposed through linear combination of a library of Raman spectra collected from purified biomolecules and fitted using the least squares method. Source data are provided in a source data folder.

Supplementary References

1. Kotta-Loizou, I. *et al.* The RNA repair proteins RtcAB regulate transcription activator RtcR via its CRISPR-associated Rossmann fold domain. en. *iScience* **25,** 105425 (Nov. 2022).
2. Monod, J., Wyman, J. & Changeux, J.-P. On the nature of allosteric transitions: A plausible model. en. *Journal of Molecular Biology* **12,** 88–118 (May 1965).
3. Palmer, T. & Bonner, P. L. in *Enzymes (Second Edition)* (eds Palmer, T. & Bonner, P. L.) 239–254 (Woodhead Publishing, Jan. 2011).
4. Cleland, W. W. Partition Analysis and the Concept of Net Rate Constants as Tools in Enzyme Kinetics^. *BIOCHEMISTRY* **14** (1975).
5. Genschik, P., Drabikowski, K. & Filipowicz, W. Characterization of the Escherichia coli RNA 3'-Terminal Phosphate Cyclase and Its σ54-Regulated Operon. *Journal of Biological Chemistry* **273.** Publisher: Elsevier, 25516–25526 (Sept. 1998).
6. Desai, K. K. & Raines, R. T. TRNA ligase catalyzes the GTP-dependent ligation of RNA with 3'-phosphate and 5'-hydroxyl termini. *Biochemistry* **51.** Publisher: American Chemical Society, 1333–1335 (Feb. 2012).
7. Scott, M., Gunderson, C. W., Mateescu, E. M., Zhang, Z. & Hwa, T. Interdependence of Cell Growth and Gene Expression: Origins and Consequences. *Science* **330.** Publisher: American Association for the Advancement of Science, 1099–1102 (Nov. 2010).
8. Shigematsu, M., Kawamura, T. & Kirino, Y. Generation of 2',3'-Cyclic Phosphate-Containing RNAs as a Hidden Layer of the Transcriptome. *Frontiers in Genetics* **9.** Publisher: Frontiers Media S.A., 562 (Nov. 2018).
9. Weiße, A. Y. *et al.* Mechanistic links between cellular trade-offs, gene expression, and growth (2015).
10. Das, U. & Shuman, S. 2'-Phosphate cyclase activity of RtcA: a potential rationale for the operon organization of RtcA with an RNA repair ligase RtcB in Escherichia coli and other bacterial taxa. *RNA* **19.** Publisher: Cold Spring Harbor Laboratory Press, 1355 (Oct. 2013).
11. Maughan, W. P. & Shuman, S. Distinct Contributions of Enzymic Functional Groups to the 2',3'-Cyclic Phosphodiesterase, 3'-Phosphate Guanylylation, and 3'-ppG/5'-OH Ligation Steps of the Escherichia coli RtcB Nucleic Acid Splicing Pathway. *Journal of bacteriology* **198.** Publisher: J Bacteriol, 1294–1304 (Apr. 2016).
12. Joly, N. & Buck, M. Single Chain Forms of the Enhancer Binding Protein PspF Provide Insights into Geometric Requirements for Gene Activation. *The Journal of Biological Chemistry* **286.** Publisher: American Society for Biochemistry and Molecular Biology, 12734 (Apr. 2011).
13. Joly, N., Zhang, N. & Buck, M. ATPase Site Architecture Is Required for Self-Assembly and Remodeling Activity of a Hexameric AAA+ Transcriptional Activator. *Molecular Cell* **47,** 484–490 (Aug. 2012).
14. Bernstein, J. A., Khodursky, A. B., Lin, P.-H., Lin-Chao, S. & Cohen, S. N. Global analysis of mRNA decay and abundance in Escherichia coli at single-gene resolution using two-color fluorescent DNA microarrays. *Proceedings of the National Academy of Sciences* **99.** Publisher: Proceedings of the National Academy of Sciences, 9697–9702 (July 2002).
15. Mempin, R. *et al.* Release of extracellular ATP by bacteria during growth. *BMC Microbiology* **13,** 301 (Dec. 2013).
16. Lasko, D. R. & Wang, D. I. C. On-line monitoring of intracellular ATP concentration in Escherichia coli fermentations. en. *Biotechnology and Bioengineering* **52,** 364–372 (1996).
17. Schneider, D. A. & Gourse, R. L. Relationship between Growth Rate and ATP Concentration in Escherichia coli: A bioassay for available cellular ATP. English. *Journal of Biological Chemistry* **279.** Publisher: Elsevier, 8262–8268 (Feb. 2004).
18. Karp, P. D. *et al.* The EcoCyc Database. en. *EcoSal Plus* **6** (ed Kaper, J.) ecosalplus.ESP–0009–2013 (May 2014).
19. Friedman, L. J. & Gelles, J. Mechanism of Transcription Initiation at an Activator-Dependent Promoter Defined by Single-Molecule Observation. *Cell* **148,** 679–689 (Feb. 2012).
20. Hughes, K. J., Chen, X., Burroughs, A. M., Aravind, L. & Wolin, S. L. An RNA Repair Operon Regulated by Damaged tRNAs. *Cell reports* **33.** Publisher: Cell Rep (Dec. 2020).

21. Fegatella, F., Lim, J., Kjelleberg, S. & Cavicchioli, R. Implications of rRNA Operon Copy Number and Ribosome Content in the Marine Oligotrophic Ultramicrobacterium Sphingomonas sp. Strain RB2256. *Applied and Environmental Microbiology* **64,** 4433–4438 (Nov. 1998).
22. Klumpp, S., Scott, M., Pedersen, S. & Hwa, T. Molecular crowding limits translation and cell growth. *Proceedings of the National Academy of Sciences* **110.** Publisher: Proceedings of the National Academy of Sciences, 16754–16759 (Oct. 15, 2013).
23. Thomas, P., Terradot, G., Danos, V. & Weiße, A. Y. Sources, propagation and consequences of stochasticity in cellular growth. *Nature Communications 2018 9:1* **9.** Publisher: Nature Publishing Group, 1–11 (Oct. 2018).
24. Neidhardt, F. C. (C. *Escherichia coli and Salmonella typhimurium : cellular and molecular biology* Second edition. eng (American Society for Microbiology, Washington, D.C, 1996).
