# Supplementary material for: Heterogeneity in responses to ribosome-targeting antibiotics mediated by bacterial RNA repair
Source: Nat Commun. 2025 Nov 11;16:9620. doi: 10.1038/s41467-025-64759-3 (PMC12606134; doi:10.1038/s41467-025-64759-3)
Supplement: Supplementary file 2 — Reporting Summary [file 41467_2025_64759_MOESM2_ESM.pdf]

nature portfolio

Corresponding author(s): Dr Andrea Yeong Weisse, Dr Christoph Engl

Last updated by author(s): Sep 2, 2025

Reporting Summary

Nature Portfolio wishes to improve the reproducibility of the work that we publish. This form provides structure for consistency and transparency in reporting. For further information on Nature Portfolio policies, see our Editorial Policies and the Editorial Policy Checklist.

Statistics

For all statistical analyses, confirm that the following items are present in the figure legend, table legend, main text, or Methods section.

n/a	Confirmed	
☐	☒	The exact sample size (*n*) for each experimental group/condition, given as a discrete number and unit of measurement
☐	☒	A statement on whether measurements were taken from distinct samples or whether the same sample was measured repeatedly
☐	☒	The statistical test(s) used AND whether they are one- or two-sided<br>*Only common tests should be described solely by name; describe more complex techniques in the Methods section.*
☒	☐	A description of all covariates tested
☒	☐	A description of any assumptions or corrections, such as tests of normality and adjustment for multiple comparisons
☐	☒	A full description of the statistical parameters including central tendency (e.g. means) or other basic estimates (e.g. regression coefficient) AND variation (e.g. standard deviation) or associated estimates of uncertainty (e.g. confidence intervals)
☐	☒	For null hypothesis testing, the test statistic (e.g. *F*, *t*, *r*) with confidence intervals, effect sizes, degrees of freedom and *P* value noted<br>*Give P values as exact values whenever suitable.*
☒	☐	For Bayesian analysis, information on the choice of priors and Markov chain Monte Carlo settings
☒	☐	For hierarchical and complex designs, identification of the appropriate level for tests and full reporting of outcomes
☒	☐	Estimates of effect sizes (e.g. Cohen's *d*, Pearson's *r*), indicating how they were calculated

Our web collection on statistics for biologists contains articles on many of the points above.

Software and code

Policy information about availability of computer code

Data collection	Matlab using Schnitzcells and Spaetzcells toolboxes, references provided in Methods.
Data analysis	Julia version 1.11: DifferentialEquations v7.15.0, ModelingToolkit v9.54.0, BifurcationKit v0.4.4, CairoMakie v0.12.18, DataFrames v1.7.0, Interpolations v0.15.1, HypothesisTests v0.11.3, GLM v1.9.0, KernelDensity v0..69

For manuscripts utilizing custom algorithms or software that are central to the research but not yet described in published literature, software must be made available to editors and reviewers. We strongly encourage code deposition in a community repository (e.g. GitHub). See the Nature Portfolio guidelines for submitting code & software for further information.

Data

Policy information about availability of data

All manuscripts must include a data availability statement. This statement should provide the following information, where applicable:

- Accession codes, unique identifiers, or web links for publicly available datasets
- A description of any restrictions on data availability
- For clinical datasets or third party data, please ensure that the statement adheres to our policy

The data supporting the findings of this study are available in this paper via the Source Data. Code for data analysis of Raman data is available via https://github.com/Engl-lab/Cellular-Raman.git. Model code is available on https://github.com/ hhindley/rtc_model.

Research involving human participants, their data, or biological material

Policy information about studies with human participants or human data. See also policy information about sex, gender (identity/presentation), and sexual orientation and race, ethnicity and racism.

Reporting on sex and gender	Not relevant as we only studied model bacterial strains in the laboratory.
Reporting on race, ethnicity, or other socially relevant groupings	Not relevant as we only studied model bacterial strains in the laboratory.
Population characteristics	Not relevant as we only studied model bacterial strains in the laboratory.
Recruitment	Not relevant as we only studied model bacterial strains in the laboratory.
Ethics oversight	Not relevant as we only studied model bacterial strains in the laboratory.

Note that full information on the approval of the study protocol must also be provided in the manuscript.

Field-specific reporting

Please select the one below that is the best fit for your research. If you are not sure, read the appropriate sections before making your selection.

☒ Life sciences ☐ Behavioural & social sciences ☐ Ecological, evolutionary & environmental sciences

For a reference copy of the document with all sections, see nature.com/documents/nr-reporting-summary-flat.pdf

Life sciences study design

All studies must disclose on these points even when the disclosure is negative.

Sample size	For RNA FISH data: since expression of rtc has previously been reported low, we chose a large number of cells (~500 in each condition, specifically N=494 for WT conditions and N=509 for conditions that were supplemented with tetracycline). We reasoned that if we assumed a defect proportion (i.e. proportion of expressing cells) of 10% and imposed a minimum precision of 5%, we would require a minimum sample size of 144. However, since the defect proportion was unknown to us, we opted for a larger sample size of roughly 500. For Raman data we collected data from at least 80 randomly selected cells per condition tested. Exact sample sizes are described in the manuscript.
Data exclusions	Fluorescent spots within segmented cells were detected and differentiated from nonspecific background signals by setting a false-positive threshold using rtcB-deficient cells as a negative control. False-positive spots were excluded by setting the threshold at the 99.9th percentile of spot intensities observed in rtcB-deficient cells. Fluorescent spots exceeding the false-positive threshold were classified as specific signals corresponding to rtcB mRNA molecules hybridised with complementary DNA probes.
Replication	All experiments were performed with at least 3 biological replicates.
Randomization	not relevant as no covariates were analysed.
Blinding	not relevant since there was no group allocation

Reporting for specific materials, systems and methods

We require information from authors about some types of materials, experimental systems and methods used in many studies. Here, indicate whether each material, system or method listed is relevant to your study. If you are not sure if a list item applies to your research, read the appropriate section before selecting a response.

Materials & experimental systems

n/a	Involved in the study	
☒	☐	Antibodies
☒	☐	Eukaryotic cell lines
☒	☐	Palaeontology and archaeology
☒	☐	Animals and other organisms
☒	☐	Clinical data
☒	☐	Dual use research of concern
☒	☐	Plants

Methods

n/a	Involved in the study	
☒	☐	ChIP-seq
☒	☐	Flow cytometry
☒	☐	MRI-based neuroimaging

Plants

Seed stocks	Not relevant as we only studied model bacterial strains in the laboratory.
Novel plant genotypes	Not relevant as we only studied model bacterial strains in the laboratory.
Authentication	Not relevant as we only studied model bacterial strains in the laboratory.
